# Fibroblast growth factor-10 and epithelial-mesenchymal transition in colorectal cancer

**DOI:** 10.17179/excli2018-1784

**Published:** 2019-07-17

**Authors:** Fatemeh Farajihaye Qazvini, Nasser Samadi, Mojtaba Saffari, Amir Nader Emami-Razavi, Reza Shirkoohi

**Affiliations:** 1 Department of Clinical Biochemistry and Laboratory Sciences, Faculty of Medicine, Tabriz University of Medical Sciences, Tabriz, Iran; 2 Group of Genetics, Cancer Research Center, Cancer Institute of Iran, Tehran University of Medical Sciences (TUMS), Tehran, Iran; 3 Iran National Tumor Bank, Cancer Institute of Iran, Tehran University of Medical Sciences, Tehran, Iran; 4 Cancer Biology Research Center, Cancer Institute of Iran, Tehran University of Medical Sciences, Tehran, Iran

**Keywords:** FGF-10, colorectal cancer, epithelial-mesenchymal transition, invasion

## Abstract

As an inducer of epithelial-mesenchymal transition (EMT), fibroblast growth factor-10 (FGF-10) has a role in cell proliferation and differentiation in the embryo in addition to invasion and metastasis during carcinogenesis. In this study, we aimed to investigate the FGF-10 gene expression in tumor tissues based on the pathological feature of tumor related to EMT and metastasis. 62 tumors were obtained from 62 colorectal cancer patients during surgery. The pathological characteristics of the patients were carefully collected and classified by Iran National Tumor Bank. To quantify FGF-10 gene expression, RNA extraction, reverse transcription-PCR and real-time PCR were respectively performed. In addition, three colorectal cancer cell lines including LS174T, SW-948 and SW-480 were collected and cultured for further molecular analysis. Consequently, FGF-10 gene expression showed increased expression level in LS174T and SW-948 while it displayed decreased level in SW-480. Considering the tumor samples, we found an upregulation of FGF-10 gene expression in 52.1 % of all tumors in stage III and only in 9.09 % of all tumors in stage I. Also, there were an upregulation of FGF-10 gene expression in 50 % of all positive lymph invasion patients. Besides, FGF-10 gene upregulation was observed in 50 % of all tumors with a size larger than 5 cm (P value < 0.05) and 69 % of all tumors located in the colon (P value < 0.05). To our knowledge, this is the first time that FGF-10 expression is reported based on pathological features of colorectal cancer.

## Introduction

Colorectal cancer is known to be a major reason behind morbidity and mortality all over the world. In fact, it is the 4^th^ cause of cancer-related deaths in the world (Haggar and Boushey, 2009[18]). It is also the 3^rd^ common cancer in men and 4^th^ common cancer in women in Iran according to the age-adjusted rate of 8.3 and 6.5 per 10^5^, respectively (Malekzadeh et al., 2009[24]). The increasing incidence of colorectal cancer in Iran during the past three decades brought it into the spotlight of research. With an overall survival rate of 41 % during a course of five years, and considerable number of young patients diagnosed with the disease compared to western countries, this has been turned into an important medical issue (Dolatkhah et al., 2015[13]). Thus, finding the factors affecting the colorectal cancer prognosis is highly critical to control the disease and increase the survival rate. 

FGF-10, a member of the FGFs family, is a mesenchymal signaling molecule in epithelium which plays a significant role during the development of several organs (Itoh, 2016[19]). In fact, this growth factor is a paracrine mediator of epithelial cell proliferation (Plichta and Radek, 2012[29]). The human FGF-10 cDNA encodes a protein including 208 amino acid (Emoto et al., 1997[15]) with 19 KD weight (Plichta and Radek, 2012[29]). This growth factor also has a role in epithelial-mesenchymal transition (EMT), wound healing and differentiation of embryonic stem cells (Itoh, 2016[19]). Furthermore, a correlation between the invasion of cancer cells and FGF-10 gene expression levels in some cancers such as prostate cancer has been reported (Abolhassani et al., 2014[1]).

EMT is as an event in which a chain of phenomena occurs starting from a change in cell-cell adhesion and cytoskeleton, followed by cell-ECM changes. This finally leads to the liberation of epithelial cells from the surrounding tissue (Shirkoohi, 2013[37]). There are three types of EMT. EMT type 1 happens during the early stages of life and is related to gastrulation and development (Shirkoohi, 2013[37]). The important role of FGF-10 in EMT type 1 has previously been reported (Abolhassani et al., 2014[1]), being vital for the appropriate development of stomach, duodenum and cecum in mice (Sala et al., 2006[33]). EMT type 2 is related to tissue regeneration and organ fibrosis, while EMT type 3 is pivotal during cancer progression and metastasis (Kalluri and Weinberg, 2009[20]). Our previous study showed that FGF-10 is effective in EMT induction through the alteration of epithelial and mesenchymal markers in breast cancer cell lines. This confirms the significance of this EMT inducer in EMT type 3 (Abolhassani et al., 2014[1]). 

It is reported that FGF-10 can increase cancer cell proliferation through enhancement of cell mitosis (Abolhassani et al., 2014[1]). Accordingly, overexpression of mitogenic growth factors may be involved in the biological aggressiveness of colorectal cancer either in a paracrine or autocrine manner (Watanabe et al., 2000[41]). The grade of tumor is categorized based on the degree of differentiation. In this sense, poorly differentiated tumors are considered as high grade which corresponds to poor patient survival (Fleming et al., 2012[16]). Clinical findings such as tumor invasion and number of nodal involvements have a direct relationship with the prognosis of colorectal cancer patients (Gunderson et al., 2010[17]). Currently, tumor staging (TNM) is believed to be the most powerful prognostic factor for colorectal cancer patients (Resch and Langner, 2013[30]). Also, it has been reported that tumor size is a potent prognosticator parameter in colon cancer patients (Kornprat et al., 2011[22]). Markers which are effective on type 3 of EMT and are accompanied with metastasis, can be good candidates for investigating the prognosis of this cancer. Our previous study has shown that FGF-10 is an inducer of EMT type 3 in breast cancer (Abolhassani et al., 2014[1]). Therefore, in this study we attempted to investigate the FGF-10 as an EMT inducer in colorectal cancer prognosis based on pathological characteristics of the tumor. Thus, we first tried to determine if there is any difference in FGF-10 expression level in colorectal cancer tumor tissues compared to normal ones. Second, we investigated whether there is any significant relationship between pathological characteristics of tumor and FGF-10 expression. To our knowledge, this is the first time that FGF-10 expression is reported based on pathological features of colorectal cancer tissues related to EMT and metastasis. 

## Materials and Methods

### Cell lines and culture situations

Three human colorectal cancer cell lines, including LS174T, SW-948, and SW-480 were obtained from Iran Genetic and Biologic Resource Center and Pasteur Institute of Iran. The colorectal cancer cell lines were cultured in RPMI-1640 containing 10 % fetal bovine serum (FBS), and also a mixture of penicillin and streptomycin to inhibit contamination. All cell lines were incubated in a humidified atmosphere comprising of 5 % CO_2_ at 37 °C. The pathological stage of cell lines includes LS174T; stage Dukes B, SW-948; stage Dukes C, and SW-480; stage Dukes B.

### Patients and samples

Colorectal cancer tissues were collected from 62 colorectal cancer patients who underwent surgical therapy at Imam Khomeini Hospital Complex and stored in Tumor bank of Cancer Institute of Iran (Tehran, Iran). Seven normal tissue samples were also included in this study. Moreover, the pathological characteristics of the patients were collected and classified carefully by Iran National Tumor Bank. The tissue samples were divided to two parts for both pathological and molecular assessments. All samples were stored at -80 °C to avoid RNA degradation. Written informed consent was received from all patients, and the scientific research ethics committee of Tehran University of Medical Sciences (Tehran, Iran) approved the protocol of this study.

### RNA isolation and reverse transcription-PCR

The tissue samples were crushed and homogenized. All RNAs of tissues and cell lines were extracted using RiboExTMLS reagent (RiboEx, GenAll, South Korea) based on manual protocol. The RNA quality was observed by electrophoresis on 1.5 % agarose gel using ethidium bromide. The 18S and 28S ribosomal RNA bands were clearly visible. Also, the evaluation of isolated RNA quantity was monitored by NanoDrop (ND-2000, USA). The RNA extracted with optimum A260/A280 ratio and the A260/A230 ratio equal to or more than 1.8 were chosen for the next step, i.e., synthesis of complementary DNA (cDNA). Reverse transcription reaction was performed from 1-2 µg of extracted RNA using Prime Script tmRT Reagent kit (TAKARA-Japan) based on kit protocol. Also, the PCR product was confirmed by electrophoresis gel and a single sharp bond was clearly observed.

### Primer design 

FGF-10 gene as an EMT inducer (Abolhassani et al., 2014[1]) was chosen as a target gene, and GAPDH (Glyceraldehyde-3-phosphate dehydrogenase) gene as a house-keeping gene was selected as a reference gene. Based on genes sequences, primers were designed using *Primer3* software. To eschew secondary structure, oligonucleotide sequences were compared and aligned with complete human genome and transcriptome. Primer sequences are shown in Table 1(Tab. 1).

### Quantitative real-time polymerase chain reaction

The real-time polymerase chain reaction was performed by SYBR®Premix ex TaqtmII (Tli RNaseH plus) (TAKARA-Japan) and Rotor-Gene Q Series Software version 2.1.0 (Build 9) (Qiagen, USA). The total volume for PCR reaction was 20 μl including 10 μl of SYBR Green PCR master mix, 2 μl of forward and reverse primers, 2 μl of cDNA template and 6 μl ddH_2_O. Real-time PCR program for the reaction was based on a holding step at 95 °C for 30 seconds, followed by 40 cycles of initiation at 95 °C for 15 seconds, annealing at 60 °C for 30 seconds, and a single final step at 58 °C for 90 seconds. In the last part of the program, the melting curve was checked and the data were evaluated by Threshold Cycle (CT) calculation using MS Excel 2013 (Microsoft, USA). Change fold of gene expression was calculated using 2^-∆∆Ct ^formula in which ∆Ct and ∆∆Ct formulas are as below. 

**ΔCt = Ct (gene of interest) - Ct (housekeeping gene)**

**ΔΔCt = ΔCt (Sample) - ΔCt (Control average)**

### Statistical analysis

For descriptive statistics in the qualitative variable, N (Percent) was reported, while in quantitative variables, Mean (SD) was stated. Independent sample T-test was used to compare a variable with two independent groups and a quantitative variable. One-way ANOVA was used to compare a variable with more than two independent groups and a quantitative variable. SPSS (version 19.0; IBM Corp., Armonk, NY) was used for statistical analysis. The statistical significance level of this study was considered as p-value < 0.05.

## Results

### Demographic data 

Regarding the stages of disease, there were four different stages including stage I (n = 11), stage II (n = 25), stage III (n = 23) and stage IV (n = 3). Tumor tissue stages were classified based on the Union for International Cancer Control (UICC) TNM classification system. Also, there were four different grades of the tumors including grade I (n = 17), grade II (n = 28), grade III (n = 8), grade IV (n = 5) and grade X (n = 4, unknown). The mean size of tumors was 5.03 ± 2.31 cm, while 40 patients had a tumor size equal to or smaller than 5 cm, and 22 patients had a tumor size larger than 5 cm. The primary site of samples was included in the colon (n = 35) and rectum (n = 26). The mean age of the patients (including 27 women and 35 men) was 65.45 ± 14.5 years old ranging from 16-80. In addition, the number of positive lymph invasion patients and negative lymph invasion patients were 28 and 32, respectively (see Table 2(Tab. 2)).

### Real-time PCR confirmation 

**Figure 1:**
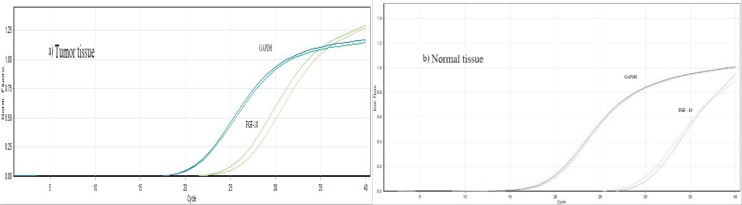
The sigmoidal shape cycles of normal and tumor tissues confirming the normal qPCR amplification curve shape

The sigmoidal shape cycles in tumor and normal tissues, confirming the normal qPCR amplification curve shape, are demonstrated in Figure 1(Fig. 1).

**Figure 2:**
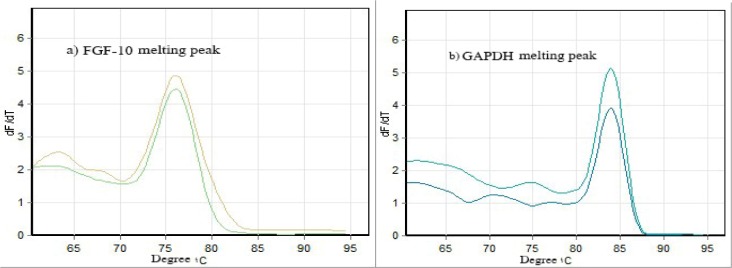
Melting curve peaks for FGF-10 and GAPDH confirming the specificity of products

To confirm the specificity of products, melting curve peaks are shown in Figure 2(Fig. 2). The melting curves show specific amplicon for FGF-10 melted at 76 °C and for GAPDH melted at 84 °C. 

### FGF-10 gene expression in cell lines and tumor tissues

**Figure 3:**
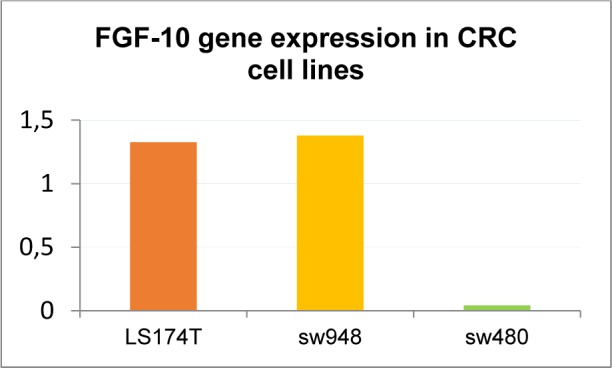
FGF-10 gene expression in colorectal cancer cell lines

Quantitative analysis showed that the FGF-10 gene expression is increased in LS174T (1.32 ± 0.72) and SW-948 (1.37 ± 0.13) while it is decreased in SW-480 (0.06 ± 0.01) (see Figure 3(Fig. 3)). 

FGF-10 gene expression in cell lines and pathological stage of cell lines are shown in Table 3(Tab. 3).

Regarding the sample tissues, FGF-10 demonstrated low gene expression (mCT = 27.80 ± 0.66) in normal colorectal tissues. The pattern of FGF-10 expression in tumor tissue based on pathological reports of the stage, grade, lymph invasion, site and size of colorectal tumors was evaluated. Our results indicated high levels of FGF-10 gene expression in 52.1 % of all tumors in stage III, while only 9.09 % upregulation was observed in tumors in stage I. Considering the grade parameter, we observed FGF-10 gene upregulation in 57.1 % of all tumors in grade II. Also, FGF-10 gene expression was elevated in 50 % of all positive lymph invasion patients. With regard to the size of tumors, FGF-10 gene upregulation was observed in 50 % of all tumors with size larger than 5 cm, and the tumor size was significantly associated with FGF-10 gene expression (P value < 0.05). Considering the site of tumors, FGF-10 gene expression showed increased levels in 69 % of all tumors located in the colon, and there was a significant correlation between FGF-10 gene expression and tumor site (P value < 0.05). There was no significant correlation between the FGF-10 gene expression and tumor stage, tumor grade, lymph invasion and age of patients (P value >0.05).

## Discussion

Colorectal cancer (CRC) is a disease in which pathogenesis is caused by genetic and epigenetic agents happening with initiation and progression of the tumor (Sinicrope et al., 2016[38]). In numerous studies, the connection of one or two molecular markers with CRC clinical characteristics, treatment result, and survival has been reported. For instance, patients in stage IV with KRAS mutation do not respond to anti-EGFR treatment (Alwers et al., 2019[2]). The most powerful prognostic parameter and determinant of therapy, applied in clinical routine, is the stage of CRC at the time of diagnosis (Alwers et al., 2019[2]). As a result, it is very important to find new markers specific for certain stages of CRC. 

The WNT, RAS-MAPK, PI3K, TGF-β, P53 and DNA mismatch-repair pathways are important in the initiation and progression of CRC (Cancer Genome Atlas Network, 2012[7]). These molecular pathways regulate several events including EMT (Rodriguez-Salas et al. 2017[31]). The EMT process produces cells with stem-like features (cancer stem cells) (Wu and Yang, 2011[42]). This indicates a link between EMT and cancer stem cells which is well documented (Wu and Yang, 2011[42]). It has been shown that stem-like cells play a rate-limiting role during tumor cell dissemination and generation of metastasis (Sampieri and Fodde, 2012[34]). Many factors are influential in EMT such as different types of growth factors (Abolhassani et al., 2014[1]). Fibroblast growth factors have important roles in organogenesis, development of tissues and also differentiation of stem cell (Chan et al., 2010[9]). FGF-10 is also effective in the embryonic period of life, during gastrulation and organogenesis (Abolhassani et al., 2014[1]). In fact, the role of FGF-10 in the development of stomach and colon (Sala et al., 2006[33]), liver (Berg et al., 2007[6]), lung (Bellusci et al., 1997[5]), and mammary glands (Cui and Li, 2013[12]) has been demonstrated. In addition, FGF-10 plays an important role in several cancers including pancreas (Nomura et al., 2008[27]), prostate (Memarzadeh et al., 2007[26]), lung (Clark et al., 2001[11]), and breast cancer (Theodorou et al., 2004[40]). Our previous study has shown that FGF-10 plays a key role in type III EMT on MCF-7 and MDA-MB231 breast cancer cell lines. Moreover, downregulation of E-cadherin gene expression in MCF-7 cell line treated with FGF-10 recombinant protein was shown. FGF-10 gene silencing in MDA-MB-231 cell line had an opposite effect on E-cadherin gene expression, clarifying the effect of this factor on cadherin epithelial factor (Abolhassani et al., 2014[1]). The E-cadherin downregulation is an important marker for the progression of invasive carcinomas, metastatic diffusion and poor clinical prognosis (Roger et al., 2010[32]). Also, the results of another study indicated that E-cadherin downregulation *in vivo* is related to the progression and metastasis of colorectal cancer (Dorudi et al., 1993[14]). In addition to these findings, our present result demonstrates that FGF-10 might correlate with tumor cell behavior; however, further studies are required to specifically determine its roles in cancer development and metastasis.

In the current study, fresh colorectal tissues were analyzed at the same time with three CRC cell lines. Cell lines were classified based on aggressiveness, and the goal was to assess the expression pattern of the target gene. Among the CRC cell lines, the highest increase in FGF-10 gene expression was seen in SW-948 (1.37 ± 0.13). The SW-948 is classified as Dukes C. In one study, the mRNA of FGF-10 was observed in colorectal cancer tumor cells, suggesting the possible role of this factor in the growth of colorectal cancer cells (Matsuike et al., 2001[25]). To our knowledge, this is the first time that FGF-10 expression is reported based on pathological characteristics of CRC (including the stage, grade, lymph invasion, tumor size and site) related to EMT and metastasis. Our results showed upregulation of FGF-10 gene expression in 52.1 % of all tumors in stage III, suggesting that FGF-10 might be a decisive factor in a subset of colorectal cancer tumors. More importantly, only 9.09 % of tumors in stage I showed FGF-10 gene upregulation. In addition, FGF-10 gene expression was elevated in 50 % of all positive lymph invasion patients. This may justify the importance of the possible role of increased FGF-10 gene expression in advanced stages as we proposed in our previous study that FGF-10 has a direct relationship with the migration (Abolhassani et al., 2014[1]). In the advanced stage of colorectal cancer (stages III or IV), the prognosis is significantly worse compared with the curable early stages (I or II) (Lee et al., 2013[23]). Therefore, as stage is considered to be the best prognostic index for individual colorectal cancer patients (Lee et al., 2013[23]; Alwers et al., 2019[2]), we focuse on the results of stage characteristic of CRC tumors. Our result, i.e. overexpression of FGF-10 gene in more than 50 % of all tumors in stage III and in 50 % of all positive lymph invasion patients, reflects the importance of this factor in both progression and targeted therapy of colorectal cancer. 

In the current study, the median size of tumors was 5 cm (ranging from 2 to 11 cm), which was considered as a cut-off value. There was an upregulation in FGF-10 gene expression in 50 % of all tumors larger than 5 cm. In fact, the tumor size was significantly associated with FGF-10 gene expression (P value < 0.05). The tumor size is reported as an important prognostic factor for colorectal cancer patients which influences the survival (Kornprat et al., 2011[22]). Moreover, we found a significant association between the expression of FGF-10 gene and tumor site (P value < 0.05). The FGF-10 gene upregulation was observed in 69 % of all tumors located in colon, suggesting the importance of FGF-10 as a risk factor for colon cancer, as the results of three large phase III randomized clinical trials have reported that the tumor location is an important factor for overall survival of patients who experienced curative surgery for colon cancer (Aoyama et al., 2017[4]). Besides, the prognostic impact of tumor size aforementioned is stronger within the colon compared to the rectum (Kornprat et al., 2011[22]), indicating the importance of our results about the size and site of tumors. 

Understanding more details of the biological phenomena of cancer will help us understand its behavior (Sharif and O'Connell, 2012[36]). For this reason, we designed our study based on the investigation of a molecular factor related to EMT to find out a possible marker or target for the future. The most popular receptor subtype for FGF-10 is FGFR2 which activates different signaling pathways inside cells (Speer et al., 2012[39]) such as PI3K-AKT or RAS-MAPK pathways (Itoh, 2016[19]) which are responsible for cell growth and proliferation (Korc and Friesel, 2009[21]; Ornitz and Itoh, 2015[28]). A case report study has been reported the FGFR2 amplification in primary colorectal cancer, suggesting that a more in-depth insight of FGFR2 amplification in colorectal tumors could find a group of CRC patients that possibly benefit from FGFR tyrosine-kinase inhibitor therapy, since it has been shown that several FGFR TKIs, including AZD4547, have *in vitro* activity against the FGFR2-amplified colorectal cell line (Carter et al., 2017[8]). This is important in terms of treatment and survival because it has been reported that pancreatic cancer patients with an elevated FGFR2 expression have a shorter survival time relative to those with low FGFR2 expression (Nomura et al., 2008[27]). FGF-10 also has been recorded to induce cell migration and invasion of pancreatic cancer cells CFPAC-1 and AsPC-1 via interaction with FGFR2-IIIb (a particular FGFR2 isoform) and by induction of MT1-MMP (membrane type 1-matrix metalloproteinase) and TGF-1b (transforming growth factor-b1) mRNA expression. The metalloproteinases, including MT1-MMP, are involved in cell invasion ability (Nomura et al., 2008[27]) and increased levels of TGF-β also decrease adhesiveness and increases the motility of cancer cells (Chao et al., 2016[10]). Moreover, FGF-10 phosphorylates GSK3b, which leads to the activation of the Wnt signaling pathway (Abolhassani et al., 2014[1]). Several studies have shown that the activation of the Wnt signaling pathway boosts transcriptional changes in order to activate EMT process in cancer (Anastas and Moon, 2013[3]). In fact, the Wnt signaling pathway induces cell migration and invasion (Sedgwick and D'Souza-Schorey, 2016[35]), implying the importance of FGF-10 in invasion and metastasis. 

In conclusion, our results in stage and positive lymph invasion of CRC suggest the possible role of FGF-10 in a subtype of colorectal cancer; however, this view should be confirmed by additional molecular studies with a larger sample size. Moreover, the significant correlation between the FGF-10 gene expression with the size and site of the tumor indicates its importance in the prognosis and survival of CRC patients. Taken together, this study proposes that FGF-10 is a critical marker for colorectal cancer targeted therapy. However, further studies with larger sample size, FGF-10 serum level analysis, and targeting this growth factor in addition to different molecules involved in FGF-10 related signaling pathways should be considered to prove this viewpoint.

## Conflict of interest

The authors declare no conflict of interest.

## Figures and Tables

**Table 1:**
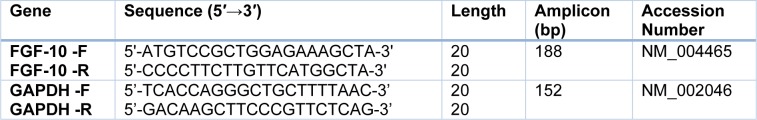
Designed primers used for quantitative RT-PCR

**Table 2:**
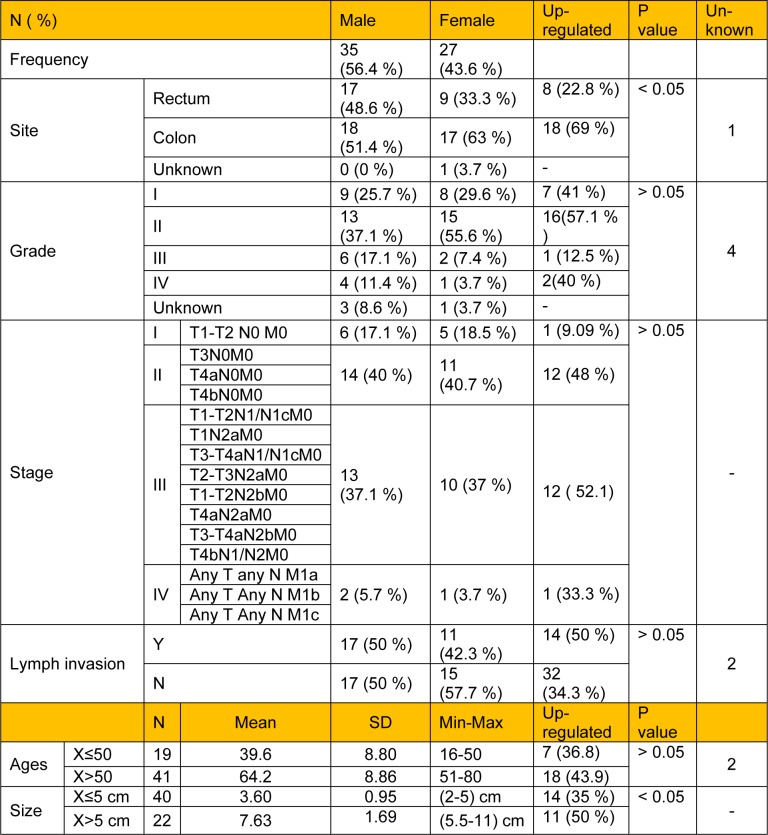
Demographic data of CRC tumors including site, stage, grade, lymph invasion, and tumor size. Stages of CRC disease were classified according to UICC TNM classification. Upregulated values of FGF-10 gene expression for all clinicopathological features of CRC tumors are also shown.

**Table 3:**
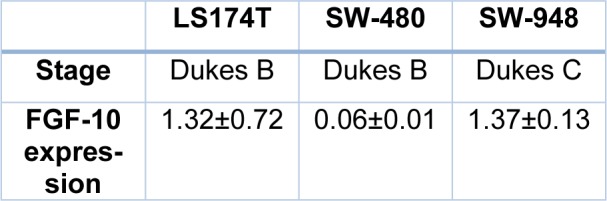
Pathological stage of colorectal cancer cell lines
